# The accuracy of preoperative implant size prediction achieved by digital templating in total knee arthroplasty is not affected by the quality of lateral knee radiographs

**DOI:** 10.1002/jeo2.12102

**Published:** 2024-07-24

**Authors:** Lorenz Pichler, Leonhard Klein, Carsten F. Perka, Clemens Gwinner, Moses K. D. El Kayali

**Affiliations:** ^1^ Charité—Universitätsmedizin Berlin Centrum für Muskuloskeletale Chirurgie Berlin Germany

**Keywords:** digital templating, knee osteoarthritis, total knee arthroplasty

## Abstract

**Background:**

Digital templating software can be used for preoperative implant size prediction in total knee arthroplasty (TKA). However, the accuracy of its prediction is reported to be low, and the impact of radiograph quality is unclear.

**Purpose:**

To investigate on the application of lateral knee radiograph quality criteria for knee rotation (KR) and knee abduction/adduction (KA) and their impact on the accuracy of final implant size prediction achieved by preoperative digital templating for TKA.

**Methods:**

A total of 191 radiographs of patients undergoing TKA were allocated into four groups according to their KR as measured at the posterior femoral condyles and their KA as measured at the distal femoral condyles on lateral knee radiographs: group A (KR ≤ 5 mm, KA ≤ 5 mm), B1 (KR > 5 mm, KA ≤ 5 mm), B2 (KR ≤ 5 mm, KA > 5 mm) and B3 (KR > 5 mm, KA > 5 mm). Preoperative templating of femoral and tibial implant size using digital templating software was carried out by two observers. Correlation coefficients (CCs) between planned and final implant size, percentage of cases with planned to final size match as well as percentage of cases within ±1 and ±2 of planned to final size were reported according to groups.

**Results:**

Group A showed the highest percentage of cases with matching planned to final femoral implant size (45%) and the highest percentage of cases with ±1 planned to final implant size (86%) as compared to B1 (match 28%, ±1 84%), B2 (match 41%, ±1 84%) and B3 (match 35%, ±1 78%). CCs for planned to final implant size were reported at >0.75 in all groups. No statistically significant difference in the CCs of planned to final implant size amongst groups was found.

**Conclusion:**

The accuracy of implant size prediction achieved by preoperative digital templating for TKA is neither affected by KR nor KA on lateral knee radiographs.

**Level of evidence:**

Level III.

**Abbreviations**
2D: two‐dimensional
3D: three‐dimensional
ICC: intraclass correlation coefficient
IQR: interquartile range
OR: operating room
SCC: Spearman's correlation coefficient
TKA: total knee arthroplasty

## INTRODUCTION

The ability to accurately predict and choose implant size in total knee arthroplasty (TKA) is important because of several reasons. First, both over‐ and undersizing of implants can have a negative impact on patient‐reported as well as functional outcomes [2, 6, 9, 11, 14]. Second, as the volume of arthroplasty procedures is projected to keep on increasing, so will the need for cost‐effective supply chain logistics and inventory management [21]. It was shown that in hip arthroplasty, preoperative three‐dimensional (3D) templating and patient‐specific instruments are able to reduce implant inventory size by 61% [7]. Preoperative 3D templating provides more accurate predictions of implant size compared to two‐dimensional (2D) templating [8, 16]. Yet, 2D preoperative templating is still considered the gold standard in arthroplasty, possibly due to its wider availability [19]. Several studies attested that 2D templating for TKA is of low accuracy in implant size prediction [3, 4, 10, 15, 17, 22]. However, there is high heterogeneity among the templating techniques used in these studies, and none of them reported on the quality of knee radiographs used for templating.

In spite of the potential benefit on patient outcome as well as implant logistics, the aim of this study is to elucidate the impact of lateral knee radiograph image quality on the accuracy of implant size prediction of preoperative 2D templating for TKA. The hypothesis put forward is that lateral knee radiographs with less than 5 mm of KR measured at the posterior femoral condyles and less than 5 mm of knee abduction/adduction (KA) measured at the distal femoral condyles show a higher percentage of cases within ±1 and ±2 of planned to final femoral and tibial implant size, respectively, as compared to radiographs with KR and KA of over 5 mm.

## MATERIALS AND METHODS

### Patients

A total of 191 patients undergoing TKA at a high‐volume academic orthopaedic surgery centre between September 2021 and March 2023 were included. 5 inclusion criteria were defined as follows: primary TKA performed on either the left or right knee, availability of preoperative knee radiographs, sufficient quality of patient records, no history of previous ligament repair surgery or osteotomies in the respective knee and patient consent. Only one model of the knee system (Attune, DePuy Synthes) with either cruciate‐retaining or posterior‐stabilized design was included. Six exclusion criteria were defined as follows: unicondylar knee arthroplasty, insufficient patient records, history of previous ligament repair surgery or osteotomies in the respective knee, absence of radiographic reference ball and the use of other total knee systems.

The data collected included demographic data (age, gender, weight, height and site), surgical data (date of surgery, implant design, femoral and tibial component size used) as well as preoperative knee radiographs. Demographic data according to sex is presented in Table 1. All cases were operated on using a standard medial approach to the knee and a tourniquet. Implant fixation was achieved by either cementation or press‐fit. The surgical technique and choice of implant size followed the surgical technique guidelines supplied by the manufacturer of the Attune total knee system exclusively used in this study. If these recommendations led to a choice between two sizes, visual inspection of the bone coverage provided by trial components of both sizes was used to choose the appropriate size.

**Table 1:** Patient demographics and surgical parameters.

	Male	Female	Overall
Cases, *n*	86 (45%)	105 (55%)	191
Age, years	68.8 (SD, 10)	72.1 (SD, 8.9)	70.6 (SD, 9.5)
Height, cm	179.8 (SD, 8.4)	163.9 (SD, 7.8)	170.9 (SD, 11.3)
Weight, kg	93.6 (SD, 17)	80.8 (SD, 16.5)	86.5 (SD, 17.9)
BMI	28.9 (SD, 4.5)	30.1 (SD, 6.0)	29.6 (SD, 5.4)
Implant design			
Cruciate retaining	64 (74.4%)	86 (81.9%)	150 (78.5%)
Posterior stabilized	22 (25.6%)	19 (18.1%)	41 (21.5%)
Narrow femoral component	3 (3.5%)	41 (39%)	44 (23%)
Final implant size			
Femoral	7 (median; IQR, 1)	5 (median; IQR, 1)	6 (median; IQR, 3)
Tibial	7 (median; IQR, 2)	5 (median; IQR, 1)	6 (median; IQR, 3)

*Note*: Values presented represent means if not stated otherwise.

Abbreviations: BMI, body mass index; IQR, interquartile range.

### Radiographs

**Figure 1:**
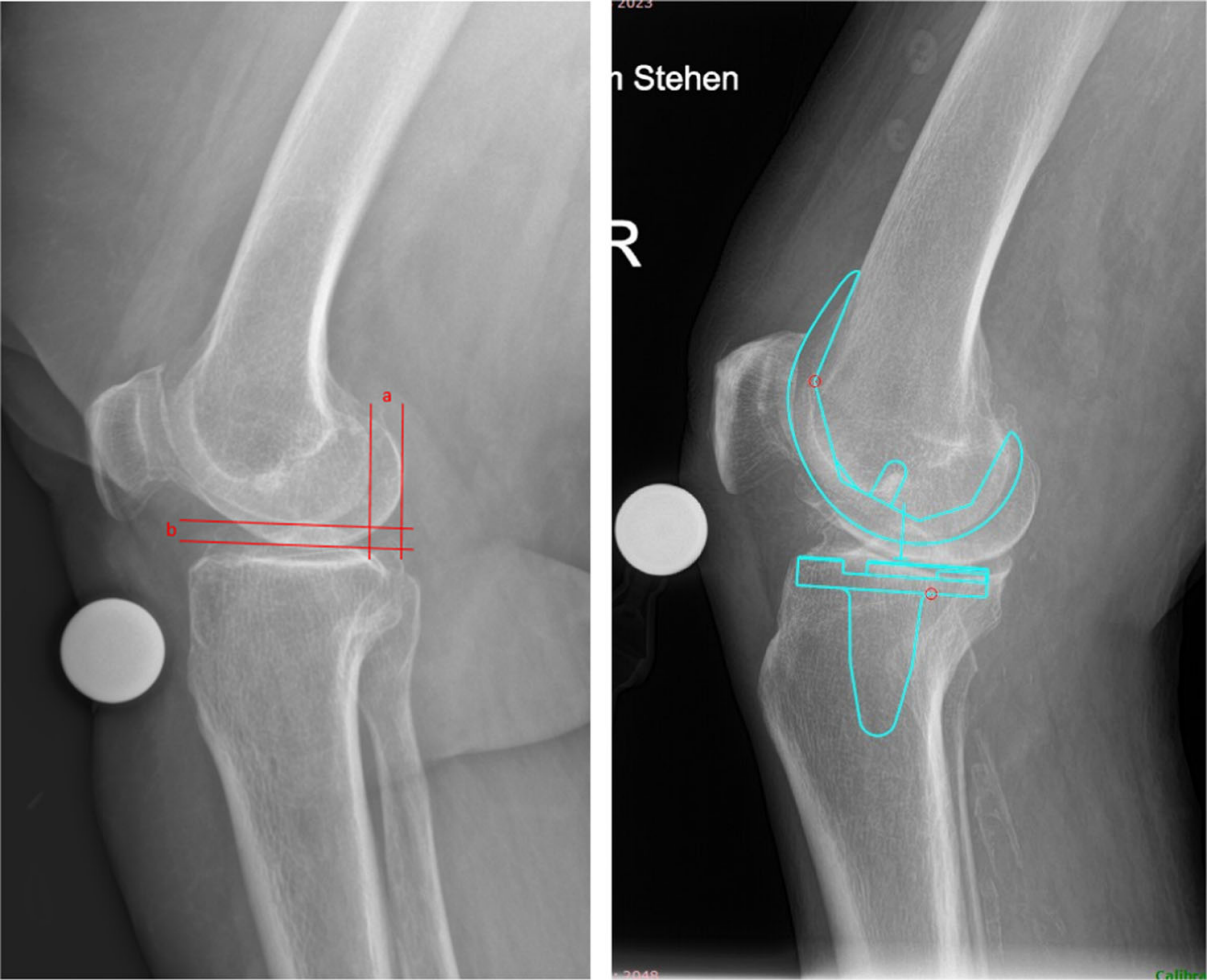
Left Lateral knee radiograph showcasing measurements of quality criteria. a—knee rotation, mm. b—knee abduction/adduction, mm. Right Lateral knee radiograph showcasing digital templating using TraumaCad (Version 2.5; Brainlab Ltd.).

Short, weight‐bearing lateral to medial and standing long‐leg anterior to posterior radiographs of the respective knee were obtained at the indication for TKA. Lateral radiographs were standardized by aiming for approximately 120° of flexion in the knee joint, the detector positioned parallel to the sagittal plane, and the centre of the radiograph aimed at the patellofemoral joint line. Long‐leg radiographs were standardized as follows: knees fully extended, feet with 10 cm distance apart and 10° external rotation, both, according to a template for feet positioning, hands alongside the body, and equal weight distribution to each leg. A standard radiographic reference ball of 25.4 mm (1 inch) diameter was used on both radiographs. Radiographs were obtained using a digital radiography system (XGEO GC85A; Samsung) at a detector‐to‐beam distance of 115 cm for lateral and 200 cm for long‐leg radiographs. For long‐leg radiographs, three separate images were obtained and automatically stitched into one via software (S‐Station, Version 3.05; Samsung). Patients were categorized into four groups according to quality criteria set for lateral radiographs, as depicted in Figure 1 and described in Table 2.

**Table 2:** Groups according to lateral knee radiograph quality criteria.

	*n*	Knee rotation (mm)	Knee abduction/adduction (mm)
Group A	90	≤5	≤5
Group B	101		
Subgroup B‐1	32	>5	≤5
Subgroup B‐2	37	≤5	>5
Subgroup B‐3	32	>5	>5

Templating was performed according to the manufacturer's guidelines using TraumaCad (Version 2.5; Brainlab Ltd.), a PACS workstation (Centricity RIS‐I 4.2 Plus; GE Healthcare), and under the guidance of a specialist for radiology, highly trained on musculoskeletal radiology. Investigations were performed by two orthopaedic surgery residents L. P. and M. E.) with 4‐ and 1‐year experience in the preoperative templating of TKA, respectively. The observers were blinded to each other's templates and carried out two separate runs of templating for each patient at different time points. Run 1 reported on the planned implant size utilizing only the lateral knee radiograph. Run 2 reported on the planned implant size utilizing both lateral and anterior‐posterior radiographs. To report on intrarater variability, each observer repeated run 2 for 15 randomly selected patients 3 weeks after the initial run 2.

### Statistics

A post hoc power analysis was carried out to report statistical power for all sample sizes tested. At a CC of 0.8 and an alpha error probability of 0.05, the power of Spearman's correlation coefficient (SCC) was calculated at 0.99 for all groups, using R (Version 4.3.2; The R Foundation). Descriptive parameters were analyzed, and mean, standard deviation of means, median and interquartile range (IQR) were calculated wherever applicable. The normality of means was assessed using the Shapiro–Wilk normality test. SCC and Kruskal–Wallis tests were performed to report on correlations and their potentially significant differences. Statistical significance was set at *p* < 0.05, and Bonferroni correction for multiple comparisons was applied where necessary.

Inter‐ and intrarater variability was assessed by calculating the intraclass correlation coefficient (ICC), which was categorized as slight (0–0.2), fair (0.21–0.4), moderate (0.41–0.6), good (0.61–0.8) or excellent (>0.8) [13]. Templating of the tibial and femoral components during runs 1 and 2 demonstrated an excellent interrater agreement at a cumulative ICC of 0.81 (*F*, 44.1; *p* < 0.001). Intrarater agreement between the initial run 2 and repeated run 2 after 3 weeks was found at an ICC of 0.85 (*F*, 57.9; *p* < 0.001) and 0.91 (*F*, 40.1; *p* < 0.001) for observers 1 and 2, respectively.

No potential bias as to the STROBE guidelines checklist [23] was found. All calculations were performed using Microsoft Excel for Mac (Version 16.7; Microsoft) and R (Version 4.3.2; The R Foundation).

## RESULTS

The distribution of implant sizes found was as follows. Femoral implant: Size 3, 2 (1%); size 4, 22 (12%); size 5, 46 (24%); size 6, 43 (23%); size 7, 38 (20%); size 8, 30 (16%); size 9, 9 (5%); size 10, 1 (1%); tibial implant: size 3, 16 (8%); size 4, 34 (18%); size 5, 43 (23%); size 6, 34 (18%); size 7, 23 (12%); size 8, 32 (17%); size 9, 7 (4%); size 10, 2 (1%). Median final implant size was found at size 7 (IQR, 1) for males and at size 5 (IQR, 1) for female patients. Categorization of patients according to lateral knee radiograph quality criteria as shown in Table 2 led to the following distribution: group A—90 patients and group B—101 patients with 32, 37 and 32 patients found for the subgroups B1, B2 and B3, respectively.

Table 3 shows the accuracy of final implant size prediction and SCCs according to the quality criteria group for both the tibial and femoral implants. Group A exhibited the highest (45%), and group B3 had the lowest percentage (35%) of cases with planned to final femoral implant size matching. The highest rate of planned to final tibial implant size matching was found in group B1 (50%) and the lowest in group B3 (40%). Run 1, using only lateral radiographs, reported lower percentages of cases within ±1 size of final implant size in all groups except one (B1, femur) when compared to run 2.

**Table 3:** Correlations of planned to final implant size according to runs.

	Group A	Group B	Subgroup B‐1	Subgroup B‐2	Subgroup B‐3	Overall
Femur						
Run 1, lateral only						
Cases planned to final size match	40%	32%	34%	35%	25%	
Cases ±1 planned to final size	77%	73%	91%	68%	63%	75%
Cases ±2 planned to final size	97%	96%	100%	100%	88%	96%
Spearman correlation coefficient	0.82*	0.82*	0.87*	0.77*	0.81*	0.82*
Run 2, lateral and long leg						
Cases planned to final size match	45%	36%	28%	41%	35%	
Cases ±1 planned to final size	86%	81%	84%	81%	78%	83%
Cases ±2 planned to final size	100%	97%	100%	97%	94%	98%
Spearman correlation coefficient	0.89*	0.88*	0.83*	0.86*	0.89*	0.88*
Tibia						
Run 1, lateral only						
Cases planned to final size match	34%	33%	33%	34%	33%	
Cases ±1 planned to final size	73%	71%	75%	73%	66%	72%
Cases ±2 planned to final size	97%	98%	100%	100%	97%	98%
Spearman correlation coefficient	0.81*	0.84*	0.89*	0.81*	0.79*	0.82*
Run 2, lateral and long leg						
Cases planned to final size match	44%	46%	50%	47%	40%	
Cases ±1 planned to final size	87%	87%	94%	89%	78%	87%
Cases ±2 planned to final size	100%	98%	100%	97%	97%	99%
Spearman correlation coefficient	0.91*	0.92*	0.95*	0.89*	0.90*	0.88*

*Note*: Calculations based on interrater means.

* Significant correlation (*p* < 0.001).

SSCs showed good to excellent correlation between planned and final implant size amongst all groups, both for the femoral and the tibial component and regardless of whether only lateral or both lateral and long‐leg radiographs were used for planning. Kruskal–Wallis test revealed no significant differences (*p* = 0.39) among quality criteria groups for the SCCs calculated for planned to final femoral and tibial implant size utilizing both lateral and long‐leg radiographs.

## DISCUSSION

The most important finding of this study was that lateral knee radiograph quality does not impact the accuracy of preoperative implant size prediction in TKA. The hypothesis put forward that lateral knee radiographs with less than 5 mm KR and less than 5 mm KA show a higher percentage of cases within ±1 and ±2 of planned to final femoral and tibial implant size, respectively, as compared to radiographs with KR and KA of over 5 mm confirmed. However, SCCs for planned to final implant size showed no statistically significant differences between groups. Therefore, it has to be assumed that the higher percentages of cases with matching planned final implant size reported for group A have been found by chance.

The agreement rate between planned and final implant size found in this study correlates to those found in similar works [17, 22] and, thereby, confirms the findings of Hsu et al. [10] that the training level of the templating surgeon does not affect the accuracy of templating. As lateral knee radiograph quality has not been proven to increase the accuracy of implant size prediction, the need for ways of doing so remains.

Apart from benefits on patient outcome, this need is driven first and foremost by the potential economic and environmental benefits of an accurate prediction of implants, and, therefore, a reduction of ‘overage’ carries [18]. In an extensive review, Ahmadi et al. [1] provided an overview of works on operating room (OR) inventory and how its active management can decrease hospital costs. Weiss et al. [24] further showed that such active management not only decreases costs but also has a positive environmental impact.

Apart from digital templating software, several other ways of implant size prediction have recently emerged. Calculating the expected implant size by only utilizing patient height, weight and gender results in a lower correlation of calculated to final implant size than preoperative templating [20]. However, by further adding patient age and body mass index and applying a machine learning approach, an algorithm alone was able to reach an accuracy almost identical to that of the presented study [12]. This further emphasizes the potential of machine learning in orthopaedics surgery.

This study carries some limitations beyond those imperative by its retrospective nature. While measurable post‐production quality criteria were applied to lateral knee radiographs, the quality of long‐leg radiographs was addressed solely through the standardization of image capturing. Further, the thresholds applied to quality criteria groups were extrapolated from those found for the measurement of the posterior tibial slope [5], and neither the manufacturer of the templating software used nor the current literature provides a framework for radiograph quality in the templating of TKA. The sole use of one total knee system has potentially decreased the risk of bias posed by different systems requiring slightly different ways of templating. However, it also limits the extrapolation of the findings of this study to other total knee systems. As lateral knee radiograph quality showed no impact on the accuracy of implant size, prediction in TKA future research should focus on further exploring ways of increasing said accuracy. Implementing an accurate and reliable way of implant size prediction carries the potential for extensive economic and environmental benefits in a modern OR setting.

## CONCLUSION

The accuracy achieved by preoperative digital templating of TKA is not affected by KR and/or KA on lateral knee radiographs. Further research on implementing accurate implant size prediction into the modern‐day arthroplasty OR is needed if the full economic and environmental potential of active implant inventory management shall be harnessed.

## AUTHOR CONTRIBUTIONS

Each named author has substantially contributed to conducting the underlying research and drafting this manuscript.

## CONFLICT OF INTEREST STATEMENT

The authors declare no conflict of interest.

## ETHICS STATEMENT

The study protocol was approved by the ethics committee of Charité—Universitätsmedizin Berlin (EA2/016/21), and the study was conducted in accordance with the Declaration of Helsinki. Written informed consent was obtained from all patients included in this study.

## Data Availability

Data that support the findings of this study are available from the corresponding author upon reasonable request.
